# Diagnostic role and immune correlates of programmed cell death-related genes in hepatocellular carcinoma

**DOI:** 10.1038/s41598-023-47560-4

**Published:** 2023-11-22

**Authors:** Zhanao He, Jie Zhang, Wukui Huang

**Affiliations:** 1 Department of Interventional Diagnosis and Treatment, The Affiliated Tumor Hospital of Xinjiang Medical University, Ürümqi, 830011 China; 2 Department of Hepatobiliary Surgery, People’s Hospital of Xinjiang Uygur Autonomous Region, Ürümqi, 830011 China

**Keywords:** Cancer, Cell biology

## Abstract

Programmed cell death (PCD) is thought to have multiple roles in tumors. Here, the roles of PCD-related genes were comprehensively analyzed to evaluate their values in hepatocellular carcinoma (HCC) diagnosis and prognosis. Gene expression and single-cell data of HCC patients, and PCD-related genes were collected from public databases. The diagnostic and prognostic roles of differentially expressed PCD-related genes in HCC were explored by univariate and multivariate Cox regression analyses. Single-cell data were further analyzed for the immune cells and expression of feature genes. Finally, we evaluated the expression of genes by quantitative real-time polymerase chain reaction and Western blot, and the proportion of immune cells was detected by flow cytometry in HCC samples. We obtained 52 differentially expressed PCD-related genes in HCC, based on which the consensus clustering analysis cluster 2 was found to have a worse prognosis than cluster 1. Then 10 feature genes were identified using LASSO analysis, and programmed cell death index (PCDI) was calculated to divided HCC patients into high-PCDI and low-PCDI groups. Worse prognosis was observed in high-PCDI group. Cox regression analysis showed that PCDI is an independent prognostic risk factor for HCC patients. Additionally, SERPINE1 and G6PD of feature genes significantly affect patient survival. Macrophages and Tregs were significantly positively correlated with PCDI. G6PD mainly expressed in macrophages, SERPINE1 mainly expressed in fibroblast. The experimental results confirmed the high expression of SERPINE1 and G6PD in HCC compared with the control, and the infiltration level of macrophages and Treg in HCC was also obviously elevated. PCDI may be a new predictor for the diagnosis of patients with HCC. The association of SERPINE1 and G6PD with the immune environment will provide new clues for HCC therapy.

## Introduction

More than one million patients will be diagnosed with liver cancer each year by 2025^1^. Hepatocellular carcinoma (HCC) accounts for approximately 75% of all primary liver cancers and is the third most common cause of cancer death worldwide^2^. Hepatitis B virus, hepatitis C virus, aflatoxins, alcohol consumption, obesity, and metabolic syndrome are the main causes of HCC^3^. HCC is a major histological type characterized by uncontrolled growth in incidence, low resection rate, and high recurrence rate after curative surgery leading to poor prognosis^4^. Insufficient early HCC detection and poor risk stratification methods, and lack of access to therapeutic agents for patients with late detected conditions may all contribute to increased HCC mortality in high-risk populations^5^. Despite the rapid progress in the detection of HCC biomarkers, the prognosis of patients remains poor, with a 5-year survival rate of less than 30%^6^. Moreover, recurrence occurs in up to 70% of cases 5 years after HCC treatment that undergo radical surgery, affecting the long-term outcome of patients^7^.

Substantial progress has been made in the development of new therapeutic means through the continuous understanding of the molecular mechanisms underlying HCC^8^. Importantly, with the implementation of targeted therapy and immunotherapy, the overall survival rate of HCC patients has been effectively improved^9,10^. Recently, several studies have revealed that the programed cell death (PCD) patterns, including apoptosis, cuproptosis, autophagy, pyroptosis, entotic cell death, parthanatos, alkaliptosis, oxeiptosis, lysosome-dependent cell death, netotic cell death, necroptosis, and ferroptosis, open new research perspectives for the treatment of cancer^11–14^. Different PCD patterns affect cancer progression and response to therapy. In addition, some PCD patterns stimulate immune responses and induce robust, long-lasting antitumor immunity^15^. Therefore, PCD patterns may be potential targets for cancer therapy, and the development of anticancer drugs by inducing or manipulating PCD may achieve more clinical benefits^16^.

In this study, differentially expressed genes of PCD in HCC were explored as prognostic factors for patients by unsupervised cluster analysis and survival analysis. Two subtypes were proposed by the least absolute shrinkage and selection operator (Lasso) algorithm and risk score, further identifying key prognostic genes. In HCC immune cell infiltration, high-level infiltration of macrophages and regulatory T cells, and correlation with prognostic genes were described and revealed. In conclusion, this work dissects the different subtypes and key genes that affect the prognosis of HCC patients from the perspective of PCD, providing help to improve the treatment strategies and prognosis of patients.

## Materials and methods

### Data collection

Clinical information and gene expression profiles of HCC patients and controls were obtained from the Cancer Genome Atlas (TCGA, https://portal.gdc.cancer.gov/) database and Gene Expression Omnibus (GEO, https://www.ncbi.nlm.nih.gov/gds) database. GSE14520 dataset^17^ in GEO database including 247 patients with HCC and 239 nontumor liver tissue samples. TCGA included 373 patients with HCC and 50 normal liver tissue samples. Somatic mutation data was also obtained from TCGA and analyzed using maftools package^18^. PCD-related genes were collected from Gene Set Enrichment Analysis (GSEA) gene set^19^. GSE149614 dataset^20^ in GEO database including single-cell RNA sequencing (scRNA-seq) transcriptomes for 10 HCC patients from primary tumor, portal vein tumor thrombus, metastatic lymph node and non-tumor liver.

### Differential expression and enrichment analysis

Genes were analyzed for differential expression between HCC and controls using limma package in R^21^, and differentially expressed genes (DEGs) were screened by *P* < 0.05 and |logFC (log fold change)|> 1. Then PCD-related genes were screened from DEGs, which were considered as differentially expressed PCD-related genes. Subsequently, we performed enrichment analysis for differentially expressed PCD-related genes using clusterProfiler package in R^22^, which including Gene Ontology (GO) analysis, and Kyoto Encyclopedia of Genes and Genomes (KEGG) signaling pathway.

### Unsupervised cluster

Next, we performed consensus clustering analysis according to the expression of differentially expressed PCD-related genes using ConsensusClusterPlus package in R^23^, which in turn classified the patients into different clusters. Overall survival (OS) of different clusters was evaluated using Kaplan–Meier (K–M) method.

### Construction of molecular subtypes

Differentially expressed PCD-related genes were used for least absolute shrinkage and selection operator (LASSO) Cox analysis in TCGA with glmnet package in R^24^. Then the prediction model of variables with non-zero coefficients was established to select the most valuable variables as feature genes using tenfold cross validation. According to expression data of feature genes, the programmed cell death index (PCDI) of each sample was calculated to construct molecular subtypes by the formula below:

$$ {\text{PCDI}} = \mathop \sum \limits_{i = 1}^{10} \beta i*Ei $$

*βi* refer to the risk coefficient and *Ei* refer to the expression of each gene. High-PCDI and low-PCDI groups of HCC patients were obtained according to the median value of the PCDI. OS of different subtypes was evaluated using K-M method. The receiver operating characteristic (ROC) curve was analyzed using survivalROC package in R, and area under the ROC curve (AUC) values were then calculated at 12-, 36-, and 60-months.

### Evaluation of PCDI and feature genes

Prognosis of PCDI was evaluated on multivariate Cox regression in GSE14520 dataset. Nomogram was structured using rms package in R^24^. Calibration curve was generated to compare consistency between predicted and actual survival probabilities. Prognosis of feature genes was evaluated on univariate Cox regression in GSE14520 dataset. Hazard ratio (HR) and 95% confidence interval (CI) were shown in forest plot. OS of feature genes was evaluated using K–M method.

### Infiltration of immune cell

The infiltration of immune cell in HCC and controls was determined using CIBERSORT method^25^. Differences in immune infiltration in HCC and controls were determined using limma package in R. Correlation between immune cells and PCDI or feature genes in HCC was calculated using Pearson correlation.

### scRNA-seq data processing

For each sample, low-quality cells with more than 8000 or fewer than 200 expressed genes were removed. The top 2000 highly variable genes were first found through the FindVariableFeatures function based on variance analysis. Clustering analysis was performed using T-distributed Stochastic Neighbor Embedding (tSNE) of Seurat. Cell types for clusters was identified by the expression of reported cell marker genes.

### Sample collection

Tumor and adjacent normal tissues of 10 HCC patients were collected during surgery from the Affiliated Tumor Hospital of Xinjiang Medical University, peripheral blood samples from HCC patients and healthy volunteers were also collected. This study was approved by the ethics committee of The Affiliated Tumor Hospital of Xinjiang Medical University (G-2021017). Written informed consent was obtained from each participant. The present study was conducted according to the 1975 Declaration of Helsinki.

### Quantitative real-time polymerase chain reaction (qRT-PCR)

To quantify the mRNA levels of feature genes, total RNA was extracted from tissue samples using TRIzol reagent (Invitrogen, CA, USA). The RNA was reverse transcribed into complementary DNA using the Takara PrimeScript™ RT-qPCR kit (Takara, Dalian, China). Then qRT-PCR was carried out using SYBR^®^ Premix Ex Taq™ II kit (Takara) on ABI 7500 detection system (Applied Biosystems, CA, USA). The following primers were used for amplification of G6PD, Forward, 5′-GCCTCAACAGCCACATGAATGC-3′ and reverse, 5′-TTCTCCACGATGATGCGGTTCC-3′; SERPINE1, Forward, 5′- AGCAGGACGAACCGCCAATC-3′ and reverse, 5′-ACAGCAGACCCTTCACCAAAGAC-3′; GAPDH, Forward, 5′-TGTTCGTCATGGGTGTGAAC-3′ and reverse, 5′-ATGGCATGGACTGTGGTCAT-3′. GAPDH was amplified as an internal control using sense primer, 5’-CGACCACTTTGTCAAGCTCA-3’ and antisense primer, 5’-GGAGAGTCAACGGGCATATAG-3’. GAPDH was used as endogenous control to calculate relative expression with 2^−∆∆Ct^ method.

### Western blot analysis

Tissues were lysed using radio immunoprecipitation assay (RIPA) lysis buffer (Beyotime, Shanghai, China) containing protease inhibitor after grinding in liquid nitrogen to extract proteins. Proteins were quantified using BCA protein assay kit (Beyotime). Equal amounts of proteins were separated by sodium dodecyl sulfate polyacrylamide gel electrophoresis. Proteins were then transferred onto polyvinylidene fluoride membranes and blocked in 5% non-fat milk for 2 h at room temperature. The membranes were incubated with the indicated primary antibodies (anti-G6PD, anti-Serpin E1, and anti-β-actin; ABclonal Technology, Wuhan, China) overnight at 4 °C. Then these membranes were incubated with the HRP-labeled secondary antibodies. Proteins were visualized using imaging system (Bio-Rad, CA, USA) with ECL chemiluminescence. Image-J software was used to calculate the relative expression normalized to β-actin protein.

### Flow cytometry assay

Peripheral blood samples were incubated with antibodies, CD4-PC7 (BD Biosciences, CA, USA), CD25-FITC (BD Biosciences), and CD127-PE (BD Biosciences) for Treg determine, and CD14-ECD (BD Biosciences), CD68-PC7 (BD Biosciences) for macrophages determine, for 15 min at room temperature. Then red blood cell lysate was added to the tube and incubated for 15 min at room temperature. After washing three times with PBS, cells were detected by flow cytometer and analyzed with Flowjo software (BD).

### Statistical analysis

Prism 7.0 (GraphPad software) and R software version 3.6.1 were used for all statistical analyses. Data were shown as the mean ± SD of triplicate experiments. Student t-test was used to detect differences. Statistical test with *P* < 0.05 were considered statistically significant.

### Ethics approval and consent to participate

This study was approved by the ethics committee of The Affiliated Tumor Hospital of Xinjiang Medical University (G-2021017). Written informed consent was obtained from each participant. The present study was conducted according to the 1975 Declaration of Helsinki.

## Results

### Differentially expressed PCD-related genes

**Figure 1:**
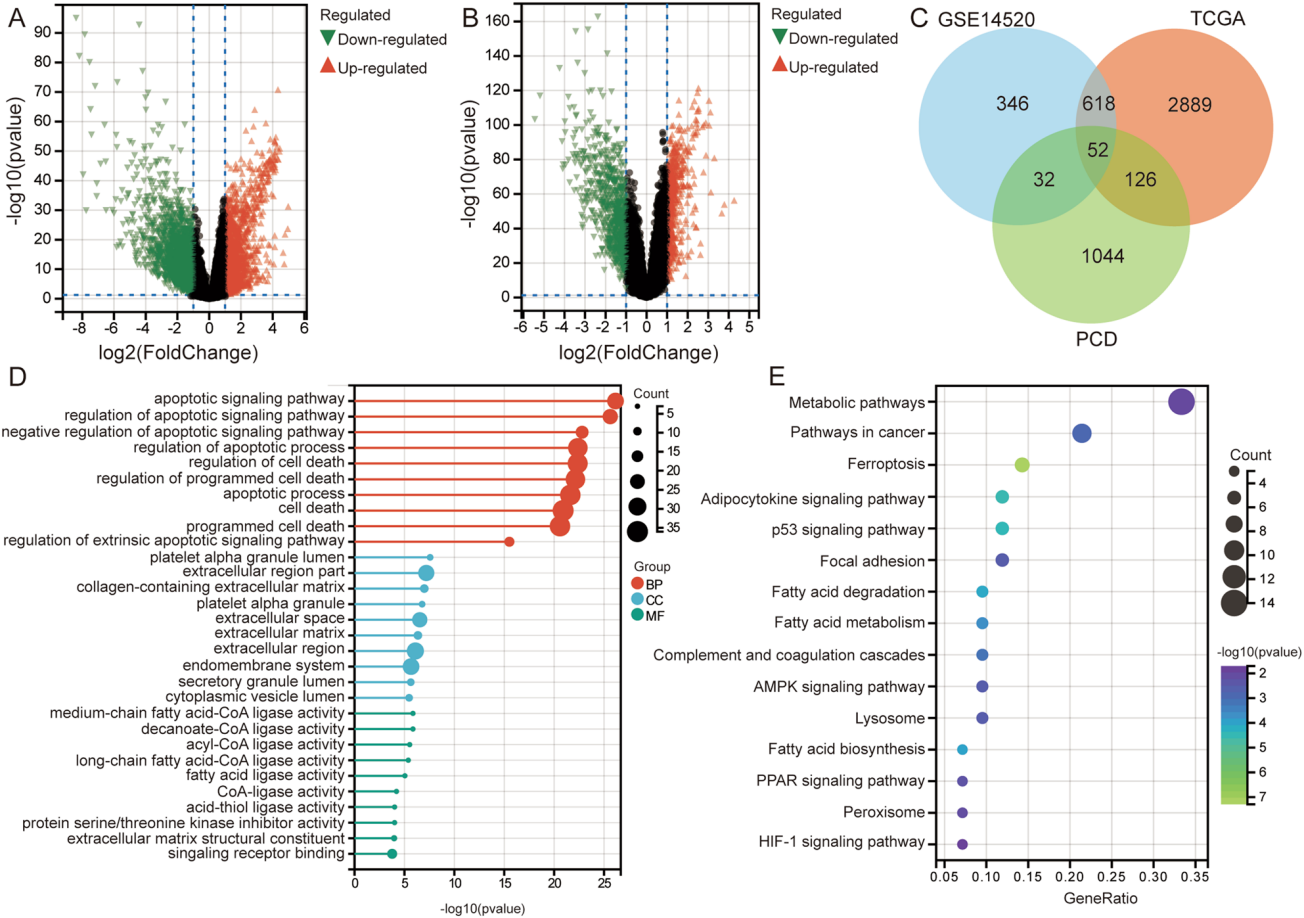
Identification of differentially expressed PCD-related genes and their biological functions in HCC. (**A**) Volcano plot of differentially expressed genes in TCGA. Red represents upregulated expression and green represents downregulated expression. (**B**) Volcano plot of differentially expressed genes in GSE14520. Red represents upregulated expression and green represents downregulated expression. (**C**) Intersection of differentially expressed genes and PCD-related genes. (**D**) Bubble plots show the main GO enrichment results related to differentially expressed PCD-related genes. (**E**) Bubble plots show the main KEGG enrichment results related to differentially expressed PCD-related genes. The larger the circle, the greater the count.

To identify differentially expressed PCD-related genes in HCC, we first performed differential expression analysis between HCC and controls. In TCGA, we identified 3685 DEGs (Fig. 1A), and in GSE14520 dataset, we identified 1048 DEGs (Fig. 1B). A total of 1254 PCD-related genes were collected from the GSEA database. Subsequently, by comparing with the DEGs in HCC, we obtained 52 differentially expressed PCD-related genes (Fig. 1C). By performing enrichment analysis on the 52 differentially expressed PCD-related genes, we mainly found apoptotic signaling pathway, regulation of cell death, and programmed cell death in the GO results (Fig. 1D). Metabolic pathways, ferroptosis, and adipocytokine signaling pathway were mainly found in KEGG results (Fig. 1E).

### Consensus clustering based on differentially expressed PCD-related genes

**Figure 2:**
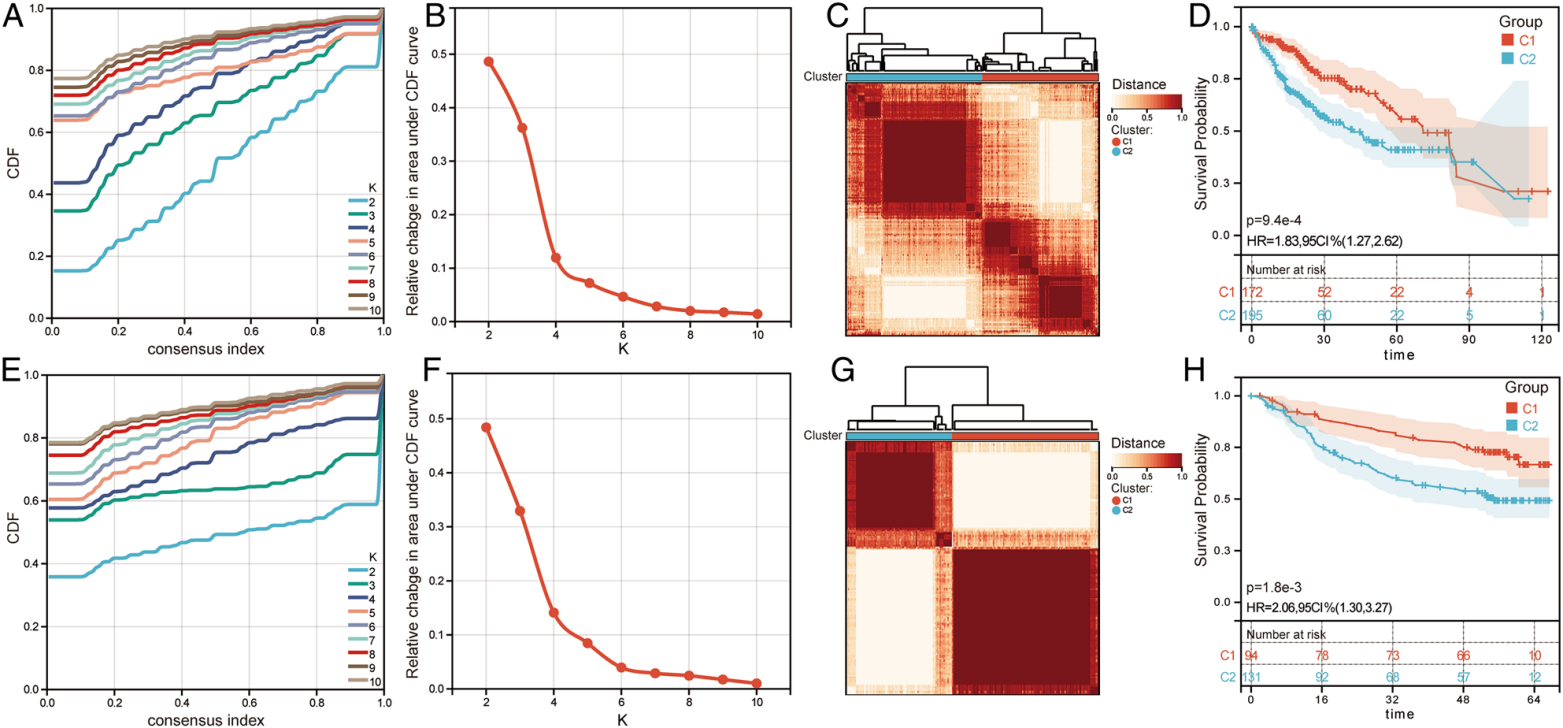
Consensus clustering analysis for HCC samples in TCGA and GSE14520 based on differentially expressed PCD-related genes. (**A**) The cumulative distribution function (CDF) with k = 2–10 in TCGA. (**B**) The area under the CDF curve for k = 2–10 in TCGA. (**C**) The distribution of consensus clustering matrix for the index k = 2 in TCGA. (**D**) Kaplan–Meier survival curves for C1 and C2 in TCGA. HR, hazard ratio; CI, confidence interval. (**E**) The CDF with k = 2–10 in GSE14520. (**F**) The area under the CDF curve for k = 2–10 in GSE14520. (**G**) The distribution of consensus clustering matrix for the index k = 2 in GSE14520. (**H**) Kaplan–Meier survival curves for C1 and C2 in GSE14520. HR, hazard ratio; CI, confidence interval.

To identify the prognostic guiding role of related genes for HCC patients, we first performed consensus clustering analysis. In the TCGA, the cumulative distribution function (CDF) of different clustering variable with k increasing from 2 to 10 is shown in Fig. 2A,B. When k = 2, two clusters with cluster 1 (C1) and cluster 2 (C2) were obtained as the best clustering stability (Fig. 2C). In the GSE14520, CDF of different clustering variable with k increasing from 2 to 10 is shown in Fig. 2E,F. When k = 2, two clusters with C1 and C2 were obtained as the best clustering stability (Fig. 2G). Survival analysis results demonstrated that HCC patients in C2 had a worse prognosis than those in C1 (Fig. 2D,H).

### Molecular subtypes based on differentially expressed PCD-related genes

**Figure 3:**
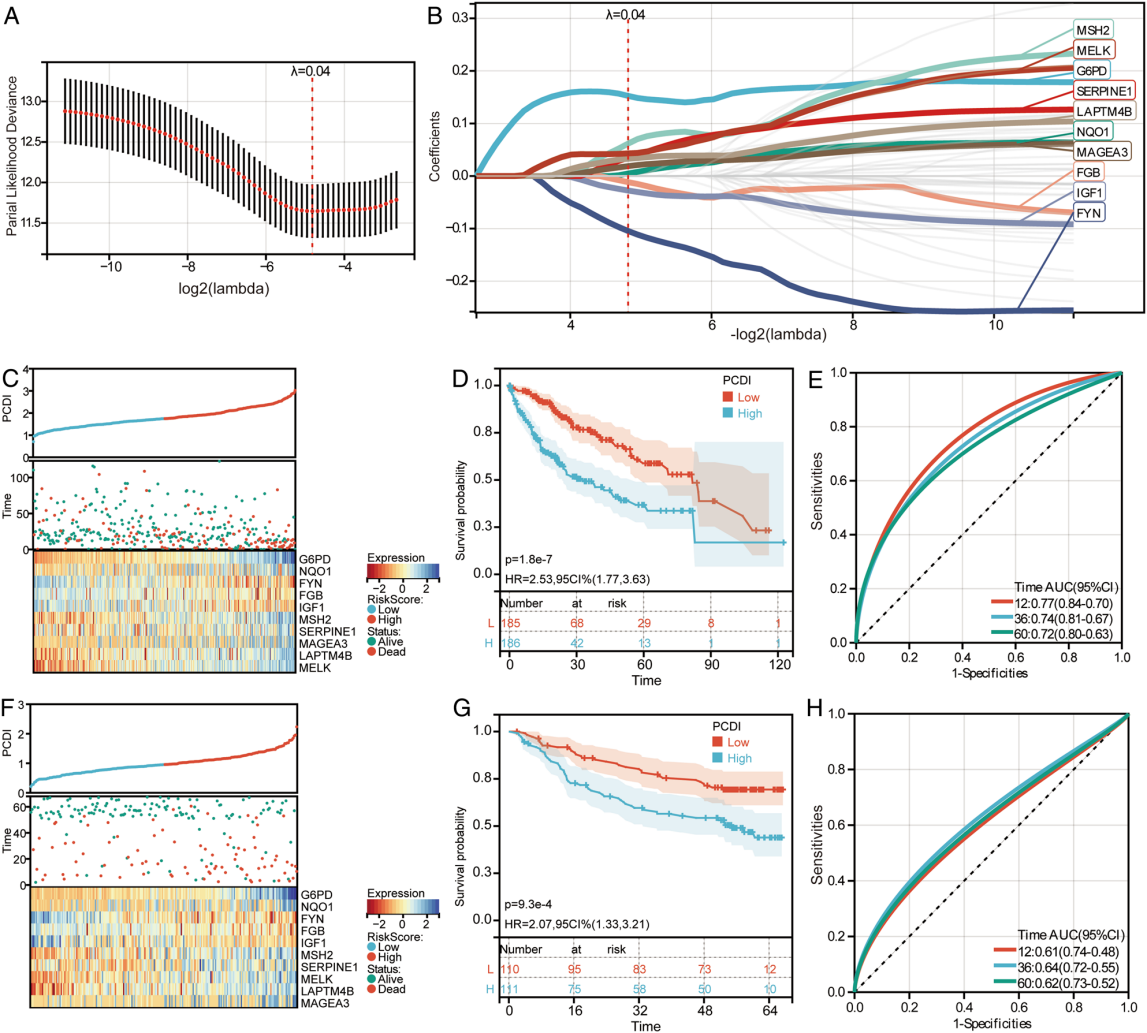
Construction of molecular subtypes based on differentially expressed PCD-related genes. (**A**) Selection of the optimal parameter (lambda) in the LASSO model in TCGA. (**B**) Lasso regression model was built in TCGA. (**C**) The HCC patients divided into high- and low-PCDI groups according to the median value of PCDI in TCGA. (**D**) Kaplan–Meier survival curves for high- and low-PCDI groups in TCGA. HR, hazard ratio; CI, confidence interval. (**E**) ROC curves of median value of PCDI for 12-, 36- and 60-month OS for HCC patients in TCGA. AUC, area under the ROC curve. (**F**) The HCC patients divided into high- and low-PCDI groups according to the median value of PCDI in GSE14520. (**G**) Kaplan–Meier survival curves for high- and low-PCDI groups in GSE14520. HR, hazard ratio; CI, confidence interval. (**H**) ROC curves of median value of PCDI for 12-, 36- and 60-month OS for HCC patients in GSE14520. AUC, area under the ROC curve.

Using LASSO analysis, we identified 10 feature genes (G6PD, NQO1, FYN, FGB, IGF1, MSH2, SERPINE1, MAGEA3, LAPTM4B, and MELK) based on the optimum λ value (λ = 0.04) in the TCGA (Fig. 3A,B). In TCGA, HCC patients were divided into high-PCDI and low-PCDI groups according to the median value of the PCDI (Fig. 3C). Survival analysis results indicated that HCC patients in high-PCDI had a worse prognosis than those in low-PCDI (Fig. 3D). ROC curves showed that 12-, 36- and 60-month AUC values for the median value of the PCDI were 0.77, 0.74 and 0.72, respectively (Fig. 3E). Additionally, In GSE14520, HCC patients were also divided into high-PCDI and low-PCDI groups according to the median value of the PCDI (Fig. 3F). Survival analysis results indicated that HCC patients in high-PCDI had a worse prognosis than those in low-PCDI (Fig. 3G). ROC curves showed that 12-, 36- and 60-month AUC values for the median value of the PCDI were 0.61, 0.64 and 0.62, respectively (Fig. 3H).

**Figure 4:**
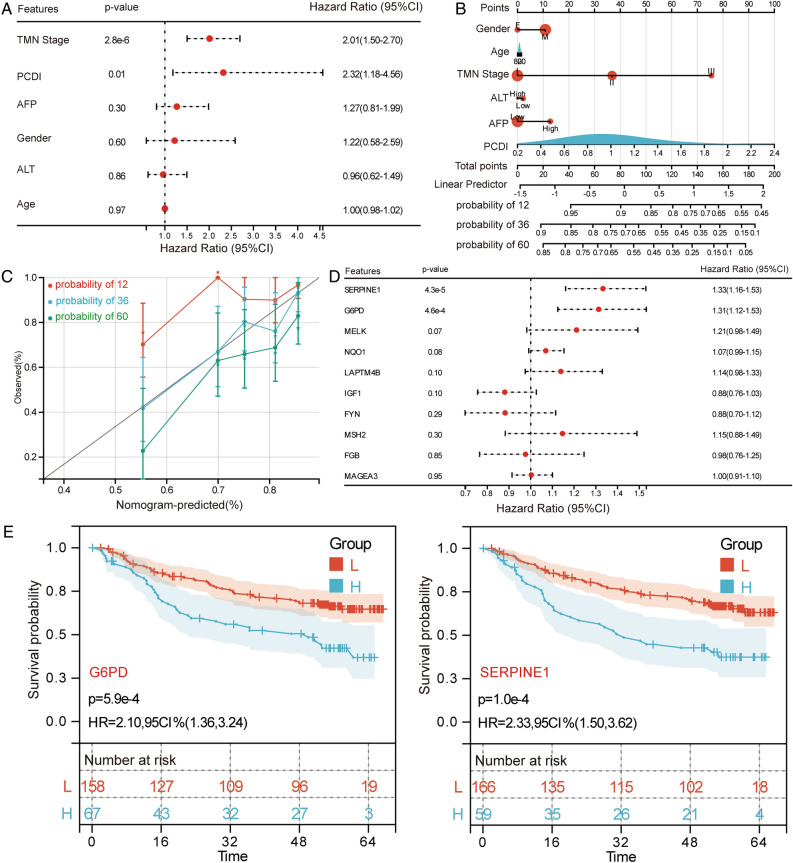
Evaluation of PCDI and feature genes. (**A**) Multivariate Cox regression analyses of PCDI and clinical factors on HCC OS in GSE14520. (**B**) Nomogram of PCDI and clinical factors for predicting 12-, 36- and 60-month OS. (**C**) Calibration curves for predicted and actual survival rates. (**D**) Univariate Cox regression analysis of feature genes on HCC OS in GSE14520. (**E**) Kaplan–Meier survival curves for SERPINE1, and G6PD in GSE14520. H, high expression; L, low expression; HR, hazard ratio; CI, confidence interval.

We performed multivariate Cox regression analysis of PCDI in HCC of GSE14520 to determine the independent predictive function. The results suggest that PCDI is a prognostic risk factor to HCC patients with hazard ratio greater than 1 (*P* < 0.05) (Fig. 4A). We then constructed a predictive nomogram using GSE14520 to evaluate the survival risk of HCC patients at 12-, 36- and 60-month (Fig. 4B). The calibration showed good agreement between the actual and the nomogram predicted results (Fig. 4C). In addition, univariate Cox regression analysis was performed for feature genes in HCC of GSE14520 and results revealed that SERPINE1, and G6PD were independent prognostic risk factors (Fig. 4D). Survival analysis results demonstrated that high expression of SERPINE1, and G6PD significant correlations with the poor OS of HCC patients (Fig. 4E).

### Immune and mutational status in molecular subtypes

**Figure 5:**
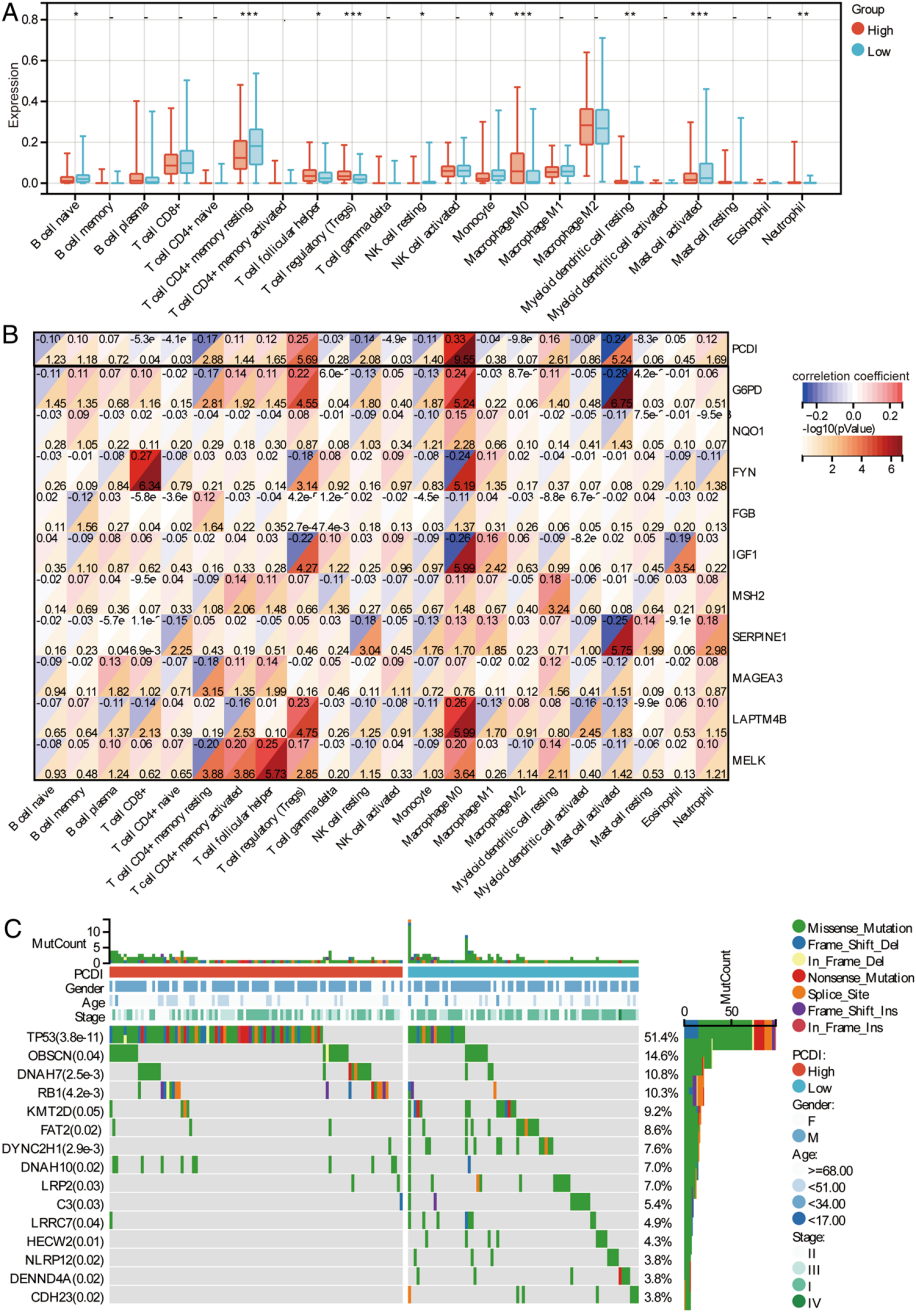
Infiltrating immune cell and somatic mutations in TCGA. (**A**) The tumour infiltration of immune cell in high-PCDI and low-PCDI groups were calculated using CIBERSORT method. *P < 0.01, **P < 0.01, ***P < 0.001. (**B**) Correlations between immune cells and feature genes. Red represents positive correlation and blue represents negative correlation. (**C**) Oncoplots of somatic mutations in high-PCDI and low-PCDI groups.

Immune status was measured using CIBERSORT method for high- and low-PCDI groups in TCGA. The results showed that infiltrated level of T cell regulatory (Tregs), macrophage M0, myeloid dendritic cell resting, and neutrophil were higher in the high-PCDI group than that in the low-PCDI group, while B cell naïve, T cell CD4+ memory resting, natural killer (NK) cell resting, monocyte, and mast cell activated were lower in the high-PCDI group (Fig. 5A). The results of correlation analysis showed that the positive correlation was highest in macrophages M0 and Tregs and PCDI (Fig. 5B). Furthermore, there was a significant positive correlation between macrophages and G6PD or SERPINE1, between Treg and G6PD or SERPINE1. In addition, we also observed the somatic mutation landscape in high-PCDI and low-PCDI groups in TCGA (Fig. 5C). It was evident that TP53 had a significantly higher mutation rate in the high-PCDI group compared to low-PCDI group.

### Integrated feature genes in the single cell level

**Figure 6:**
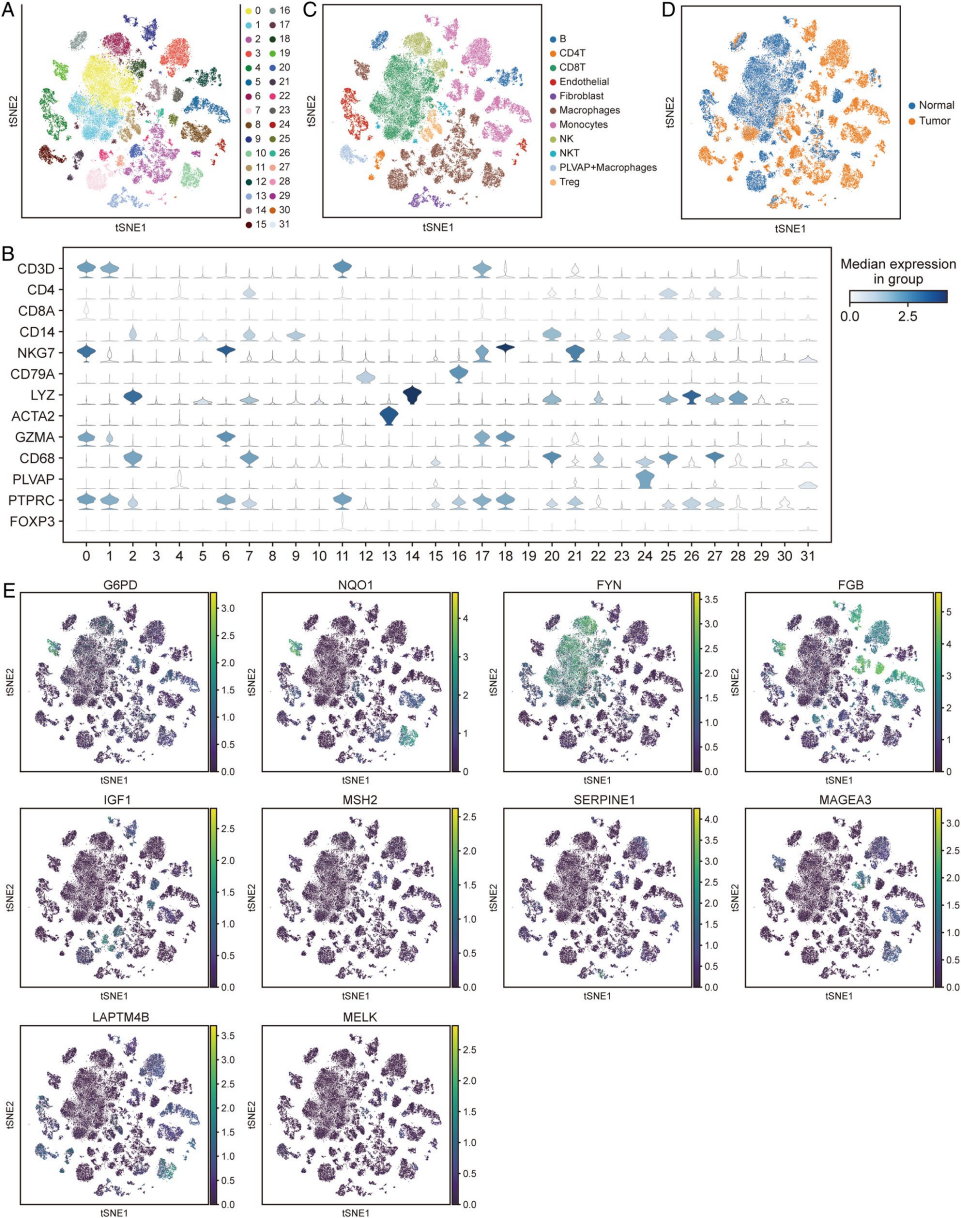
scRNA-seq of cell subsets and feature genes expression in HCC. (**A**) The t‐SNE plots of cells clustering. (**B**) The plot of average expression of specific marker genes in HCC and normal samples. (**C**) The t-SNE plots of major cell-type annotation according to marker gene expression. (**D**) The t-SNE plots of major cell subsets in HCC and normal samples. (**E**) Expression of feature genes in cell subsets.

To further explore the cell subsets of HCC patients and the expression profiles of feature genes in cells, we examined the scRNA-seq of GSE149614. Cells from HCC and control samples segregated into distinct cell populations, with individual cells clustered into 32 distinct cell clusters using tSNE clustering (Fig. 6A). Subsequently, we detected expression of specific marker genes from individual cell populations to suggest cell type identification (Fig. 6B). Finally, we identified 11 major cell types, including B cells, CD4 T cells, CD8 T cells, endothelial cells, fibroblast, macrophages, monocytes, NK cells, NKT cells, plasmalemma vesicle-associated protein (PLVAP) + macrophages, and Treg (Fig. 6C). Interestingly, majority of NK cells, and CD8 T cells were expressed in normal samples, majority of CD4 T cells, Treg, fibroblast, macrophages, PLVAP + macrophages, and monocytes, were expressed in HCC samples (Fig. 6D). G6PD was mainly expressed in macrophages, and monocytes, SERPINE1 was mainly expressed in fibroblast, and monocytes (Fig. 6E).

### Validation experiments

**Figure 7:**
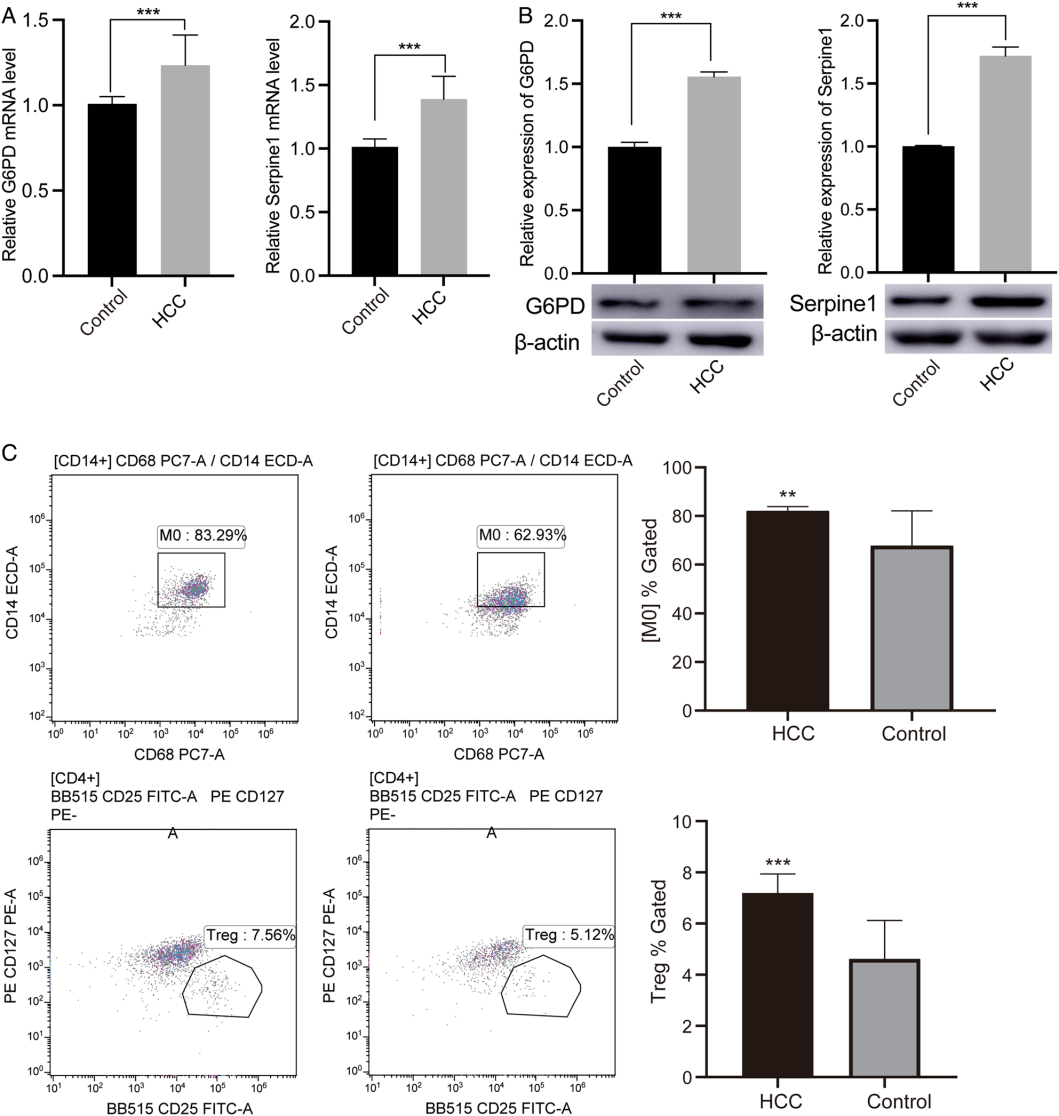
The levels of genes and cells were verified by experiments. (**A**) Relative mRNA levels of G6PD and SERPINE1 in HCC and controls were verified by qRT-PCR. (**B**) Relative expression of G6PD and SERPINE1 in HCC and controls were verified by western blotting. Original blots are presented in Supplementary Fig. 1 (**C**) The proportion of macrophages M0 and Treg were verified by flow cytometry. **P < 0.01; ***P < 0.001. *HCC* hepatocellular carcinoma.

To verify the accuracy of key results, we utilized samples from HCC patients for molecular experiments. First, we measured the mRNA levels of G6PD and SERPINE1 by qRT-PCR and found that they were all elevated expression in HCC patients compared to controls (Fig. 7A). The protein level of G6PD and SERPINE1 was detected by Western blot. The results showed that expression of G6PD and SERPINE1 was higher in HCC and control (Fig. 7B). Then, the proportion of macrophages M0 and Treg was detected by flow cytometry and showed elevated in HCC (Fig. 7C).

## Discussion

Cell death could be an attractive therapeutic approach to limit tumor growth for patients with advanced HCC^26^. As is well known, alpha fetoprotein is the most widely used serum biomarker for HCC detection and treatment evaluation. However, it is not a strong specific biomarker^27^. In addition, although a large number of studies have identified potential biomarkers and therapeutic targets for HCC, there is a lack of precise validation in clinical trials^28,29^. Further exploration of novel therapeutic strategies that target cell death and the immune microenvironment is warranted. Here, we utilized public data for an in-depth analysis of the role of PCD-related genes in the diagnosis and prognosis of HCC patients. The present study further validated gene expression and immune cell infiltration differences in human samples, suggesting that a potential link between cell death and the immune microenvironment may be an important mechanism for targeted therapy of HCC.

The most important contribution of this study was the identification and evaluation of PCD associated with HCC, and three key points were generated: (1) We describe the biological roles involved in PCD-related genes in HCC; (2) We obtained two molecular subtypes based on the expression of PCD-related genes; (3) We identified a potential link between PCD-related genes and immune cells at the single-cell level.

PCD plays an important role in homeostasis. Evasion of PCD is closely associated with clinically aggressive cancers characterized by early metastasis^30^. An increasing number of studies have confirmed that the regulation of PCD is highly beneficial for improving the survival rate of HCC patients. Here, through enrichment analysis of differentially expressed PCD-related genes, we identified multiple biological pathways. In recent decades, chemotherapy targeting apoptosis has emerged and achieved remarkable achievements^31^. Since the liver plays a key role in iron metabolism, ferroptosis plays an important role in HCC carcinogenesis and, more importantly, is effective in the treatment of HCC^32^. Adipocytokine mediates the regulation of multiple organs and tissues, bioactive molecules that elicit pleiotropic effects, including modulation of angiogenesis, metabolism and inflammation^33^. The interaction between adipocytokine and tumor cells provides pro-inflammatory factors to the tumor microenvironment, which promotes tissue damage, mutagenesis, invasion and metastasis^34^.

Based on the consensus clustering results, we identified two independent subtypes. These two subtypes were significantly different OS of patient. Next, 10 feature genes were selected by the LASSO model for constructing a novel signal to predict the OS of HCC patients. Our results revealed higher PCDI with worse prognosis in HCC patients, which may be related to the fact that most feature genes are risk genes for HCC. In addition, multivariate Cox regression analysis and nomogram results further indicated that PCDI had an advantage in predicting OS of HCC patients compared with most clinicopathological characteristics. These results suggest that the prediction model based on feature genes has high accuracy and may be a new biomarker for HCC.

In addition, glucose-6-phosphate dehydrogenase (G6PD) and serpin family E member 1 (SERPINE1) were found to be risk factors for OS in HCC patients from the univariate Cox regression analysis results. HCC patients with high expression of G6PD and SERPINE1 had a worse prognosis. Aberrant activation of G6PD leads to enhanced cell proliferation and fitness in HCC by maintaining intracellular redox homeostasis [31500396]. Single cell results suggest that G6PD is expressed in macrophages, which may be involved in tumorigenesis, drug resistance, and metastasis through metabolic reprogramming^35,36^. In fact, G6PD is highly expressed in liver cancer cells, promotes hepatocyte proliferation and tumor formation, and negatively correlates with patient survival^37^. SERPINE1 is thought to play an important role in tumor progression and angiogenesis^38,39^, which may be related to the high expression of SERPINE1 in fibroblast found in single-cell results. Upregulation of SERPINE1 promotes HCC progression^40^. Importantly, in clinical samples, we verified that G6PD and SERPINE1 were expressed higher in the tumor tissues of HCC patients than in control samples. Therefore, G6PD and SERPINE1 may become a new potential diagnostic and therapeutic target to hinder HCC progression.

In addition, PCD also regulates the enrichment of immune cells, thereby participating in the fine-tuning of antitumor immunity in the tumor microenvironment (TME)^41^. Among the significantly aberrantly infiltrated immune cells in HCC, we found that macrophages and Treg were significantly and positively correlated with PCDI. There are studies confirming that macrophages in tissues can provide a tumorigenic microenvironment for cancers in early-stage^42^. Tumor associated macrophages (TAMs), the most abundant infiltrating immune cells in the tumor microenvironment, exert a wide range of functions in HCC^43^. Macrophage populations are positively correlated with angiogenesis and poor patient prognosis in HCC^44^. In addition, TAMs can utilize chemokines to attract Treg cells to the tumor site, mediating immune tolerance of TME^45,46^. Consistent with the findings of the present study, Treg were significantly increased in the peripheral venous blood of HCC patients compared to healthy controls^47^. Treg play an important role in HCC progression by limiting the development and activation of antitumor effector cells and promoting tumor immune escape^48^. Tumor immune microenvironment can influence tumor progression and survival. At the single-cell level, we found heterogeneous clustering of immune cell type composition. Consistent with the results of transcript level analysis, macrophages and Tregs significantly clustered in HCC patients.

The present study also has limitations. First, the data we analyzed were from public databases with missing clinical information on patients, which also resulted in missing key outcomes that are also clinically relevant. Second, the relationship of gene expression and immune abnormalities should be verified and deeply explored by in vitro and in vivo experiments in future studies. Furthermore, the value of PCDI will need to be assessed in a larger cohort.

## Conclusion

In conclusion, our study confirms that PCD-related genes can affect the prognosis of HCC patients. Molecular subtypes established by feature genes based on the LASSO model have diagnostic and predictive value for the prognosis of patients with HCC. High expression of G6PD and SERPINE1 represents a poor prognosis for HCC patients and is significantly correlated with the infiltration levels of macrophages and Treg. The results of our analysis contribute to HCC patient stratification and provide new strategies for the management of cancer.

### Supplementary Information

Supplementary Figure S1.

## Data Availability

The datasets presented in this article are available in the Cancer Genome Atlas (TCGA, https://portal.gdc.cancer.gov/) database, Gene Expression Omnibus (GEO, https://www.ncbi.nlm.nih.gov/gds) database (GSE14520, and GSE149614 datasets), or from the corresponding author on reasonable request.
