# Supplementary material for: Diagnostic role and immune correlates of programmed cell death-related genes in hepatocellular carcinoma
Source: Sci Rep. 2023 Nov 22;13:20509. doi: 10.1038/s41598-023-47560-4 (PMC10665317; doi:10.1038/s41598-023-47560-4)
Supplement: Supplementary file 1 — Supplementary Figure S1. [file 41598_2023_47560_MOESM1_ESM.pdf]

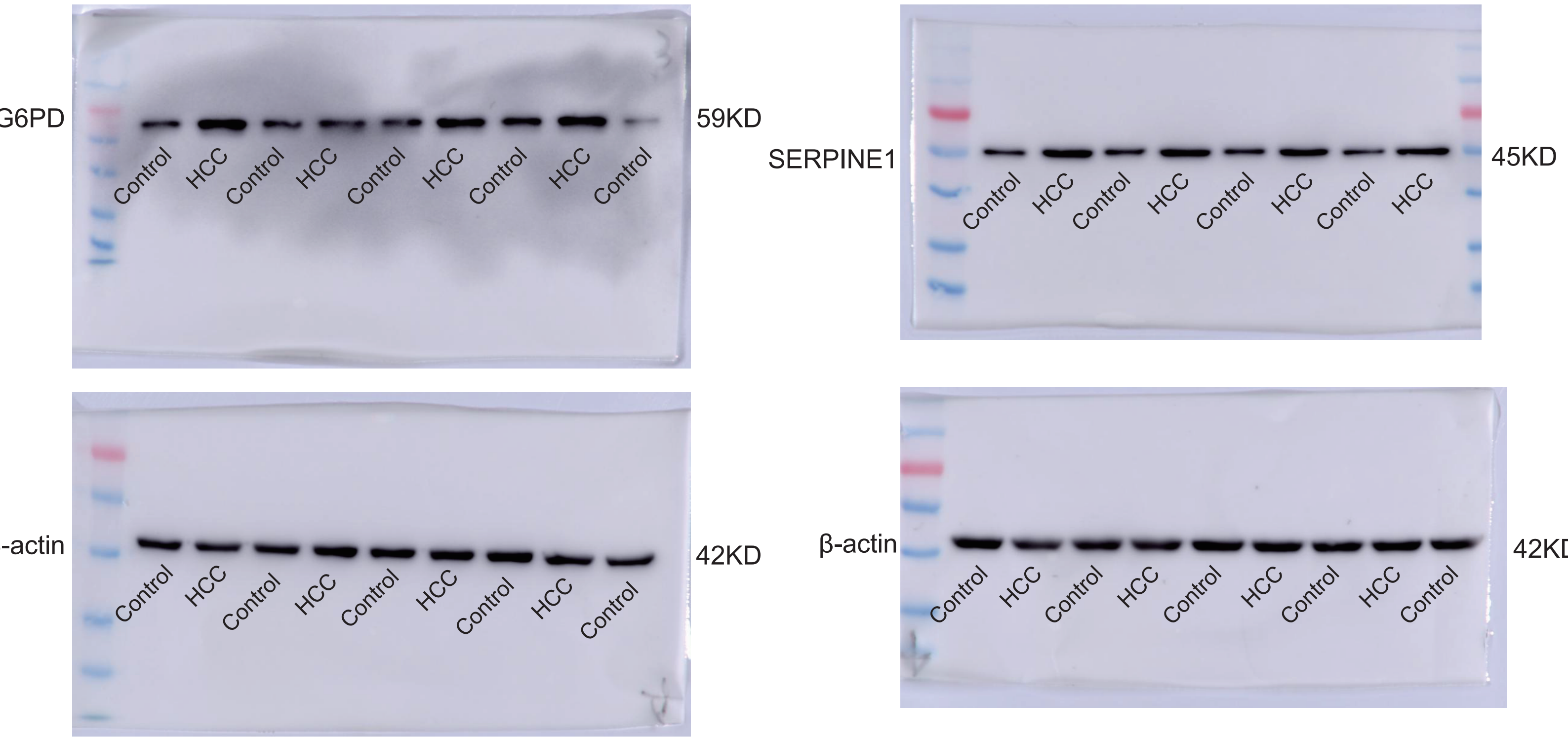

Figure S1. Images of the protein levels of G6PD and SERPINE1 detected by Western blot in HCC and controls.
